# P-2366. Influenza-Specific Antiviral Use in Hospitalized Children Before and During the COVID-19 Pandemic, New Vaccine Surveillance Network (2016–2023)

**DOI:** 10.1093/ofid/ofae631.2516

**Published:** 2025-01-29

**Authors:** Olla Hamdan, Justin Z Amarin, James W Antoon, Tess Stopczynski, Laura S Stewart, Eileen J Klein, Janet A Englund, Geoffrey A Weinberg, Peter G Szilagyi, John V Williams, Marian G Michaels, Julie A Boom, Leila C Sahni, Mary A Staat, Elizabeth P Schlaudecker, Jennifer E Schuster, Rangaraj Selvarangan, Christopher J Harrison, Ariana Perez, Heidi L Moline, James Chappell, Andrew J Spieker, Samantha M Olson, Natasha B Halasa

**Affiliations:** Vanderbilt University Medical Center; Division of Pediatric Infectious Diseases, Nashville, Tennessee; Vanderbilt University Medical Center, Nashville, Tennessee; Vanderbilt University Medical Center, Nashville, Tennessee; Vanderbilt University Medical Center, Nashville, Tennessee; Vanderbilt University Medical Center, Nashville, Tennessee; University of Washington School of Medicine, Seattle, Washington; Seattle Children’s Hospital, Seattle, Washington; University of Rochester School of Medicine & Dentistry, Rochester, NY; UCLA School of Medicine, Agoura Hills, California; University of Pittsburgh, Pittsburgh, Pennsylvania; UPMC Children's Hospital of Pittsburgh, Pittsburgh, Pennsylvania; Texas Children’s Hospital, Houston, Texas; Baylor College of Medicine and Texas Children’s Hospital, Houston, Texas; Cincinnati Children’s Hospital Medical Center, Cincinnati, Ohio; Cincinnati Children's Hospital Medical Center, Cincinnati, Ohio; Children’s Mercy Kansas City, Kansas City, Missouri; Children’s Mercy Kansas City, Kansas City, Missouri; Children's Mercy Hospital, Kansas City, Missouri; CDC, Avondale Estates, Georgia; Centers for Disease Control and Prevention, Atlanta, Georgia; Vanderbilt University Medical Center, Nashville, Tennessee; Vanderbilt University Medical Center, Nashville, Tennessee; Centers for Disease Control and Prevention, Atlanta, Georgia; Vanderbilt University Medical Center, Nashville, Tennessee

## Abstract

**Background:**

Influenza-specific antivirals are recommended for all hospitalized children with suspected or confirmed influenza. However, antiviral use remains suboptimal. We investigated antiviral use in children hospitalized with influenza before the COVID-19 pandemic and during the late pandemic period, as well as factors associated with antiviral use.

**Figure:**
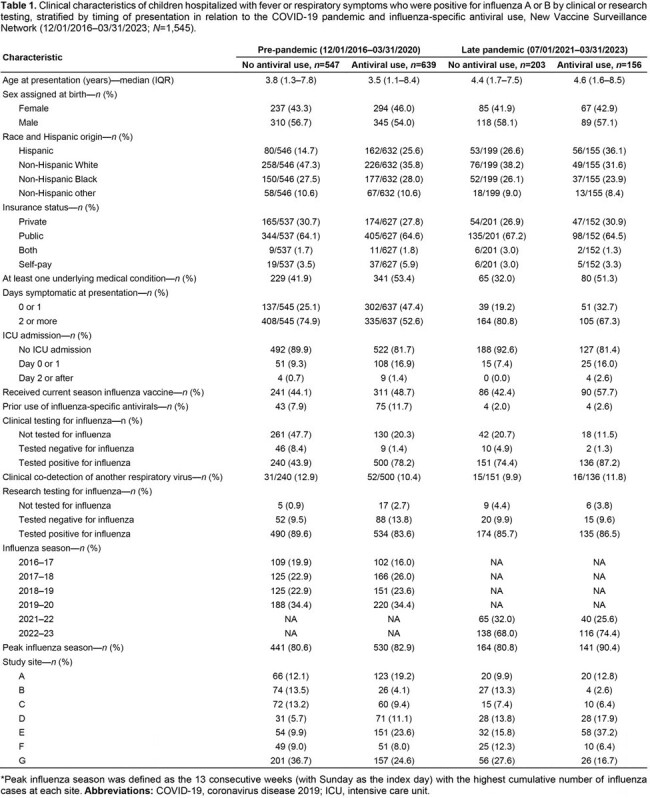

**Methods:**

We conducted active surveillance on children < 18 years old with acute respiratory illness at seven pediatric centers in the New Vaccine Surveillance Network before the COVID-19 pandemic (12/01/2016–03/31/2020) and during the late pandemic period (07/01/2021–03/31/2023). We included children hospitalized within 10 days of symptom onset who tested positive for influenza A or B viruses by clinical or research testing. We used mixed-effects Poisson regression to compare incidence proportions of antiviral use in the 2021–22 and 2022–23 seasons to before the pandemic. In addition, we fit a mixed-effects logistic regression model with the study site as a random effect to determine factors associated with antiviral use during the late pandemic period.

**Figure:**
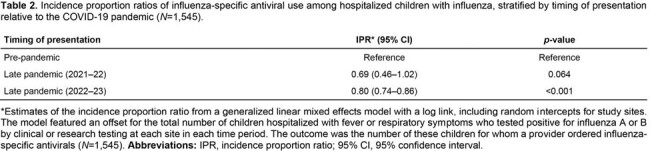

**Results:**

Among 1,549 children hospitalized with influenza, antiviral use ranged between 48.3% and 57.0% pre-pandemic but declined to 38.1% in 2021–22 and 45.7% in 2022–23 (Table 1; Figure 1). Influenza-specific antiviral use was estimated to be 31% lower in 2021–22 (*p*=0.064) and 20% lower in 2022–23 (*p*< 0.001) compared to the pre-pandemic period (Table 2), despite an increase in clinical testing. During the late pandemic, factors associated with higher odds of antiviral use included an underlying medical condition, influenza vaccination, clinical influenza testing, intensive care unit (ICU) admission, admission during the peak of influenza season, and some study sites (Figure 2).

**Figure 1.**
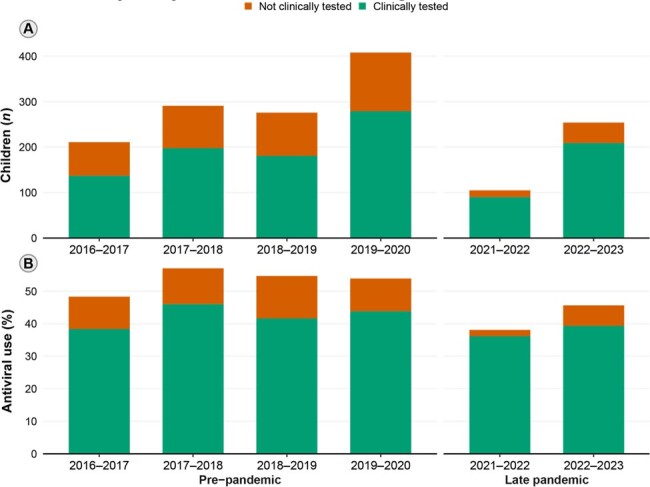
Children hospitalized within 10 days of onset of fever or a respiratory symptom who were positive for influenza A or B by clinical or research testing, disaggregated by clinical testing status, New Vaccine Surveillance Network (12/01/2016–03/31/2023; N=1,545). (A) Absolute frequencies of children hospitalized during each time period. (B) Relative frequency of antiviral use among all these children.

**Conclusion:**

Influenza-specific antiviral use in hospitalized children remained suboptimal, with a significant decline during the late COVID-19 pandemic. Our study highlights the need for enhanced efforts to improve antiviral use in this population at increased risk of severe influenza illness. Multiple factors were associated with antiviral use, underscoring the importance of considering these factors when developing interventions to optimize pediatric influenza management.

**Figure 2.**
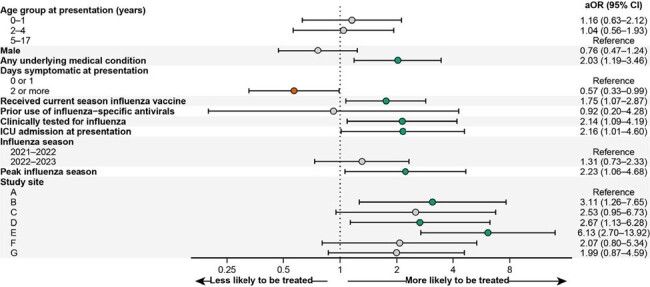
Factors associated with antiviral use among children hospitalized with influenza during the late COVID-19 pandemic period (07/01/2021–03/31/2023) in the New Vaccine Surveillance Network. All results are from a single generalized linear mixed-effects model with the study site as a random effect. Red denotes lower odds of antiviral use, and green denotes higher odds of antiviral use. Abbreviations: aOR, adjusted odds ratio; CI, confidence interval.

**Disclosures:**

James W. Antoon, MD, PhD, MPH, AstraZeneca: Advisor/Consultant|NIH: Grant/Research Support Janet A. Englund, MD, Abbvie: Advisor/Consultant|AstraZeneca: Advisor/Consultant|AstraZeneca: Grant/Research Support|GlaxoSmithKline: Advisor/Consultant|GlaxoSmithKline: Grant/Research Support|Meissa Vaccines: Advisor/Consultant|Merck: Advisor/Consultant|Pfizer: Board Member|Pfizer: Grant/Research Support|Pfizer: Speaker at meeting|SanofiPasteur: Advisor/Consultant|Shinogi: Advisor/Consultant Geoffrey A. Weinberg, MD, Inhalon: Advisor/Consultant|Merck & Company: Honoraria for textbook chapter preparation Mary A. Staat, MD, MPH, Cepheid: Grant/Research Support|Merck: Grant/Research Support|Pfizer: Grant/Research Support|Up-To-Date: Honoraria Elizabeth P. Schlaudecker, MD, MPH, Pfizer: Grant/Research Support|Sanofi Pasteur: Advisor/Consultant Rangaraj Selvarangan, BVSc, PhD, D(ABMM), FIDSA, FAAM, Abbott: Grant/Research Support|Abbott: Honoraria|BioMerieux: Grant/Research Support|Cepheid: Grant/Research Support|Diasorin: Grant/Research Support|GSK: Advisor/Consultant|Hologic: Grant/Research Support|Luminex: Grant/Research Support|Qiagen: Grant/Research Support Christopher J. Harrison, MD, GSK: Grant/Research Support|Medscape: Honoraria|Merck: Grant/Research Support|Pfizer: Grant/Research Support|UpToDate: Honoraria James Chappell, MD, PhD, Merck: Grant/Research Support Natasha B. Halasa, MD, MPH, Merck: Grant/Research Support

